# Roadmap to Creating a Global Health Equity Training Program Within US Anesthesiology Residency Programs

**DOI:** 10.1002/wjs.70212

**Published:** 2026-01-27

**Authors:** Betelehem M. Asnake, Maziar M. Nourian, Ana M. Crawford, Bantayehu Sileshi, Sheida Tabaie

**Affiliations:** ^1^ Department of Anesthesiology and Perioperative Medicine University of California Los Angeles, California USA; ^2^ Department of Anesthesiology Perioperative and Pain Medicine Stanford University Stanford California USA; ^3^ Department of Anesthesiology Vanderbilt University Medical Center Nashville Tennessee USA; ^4^ Department of Anesthesiology Weill Cornell Medicine New York New York USA

**Keywords:** anesthesiology, global health equity, residency, training program

## 1 Introduction

Global health equity education in U.S. anesthesiology residencies is gaining attention due to major global disparities in access to safe anesthesia care. The field focuses on ensuring fair access to safe effective anesthesia—especially in resource‐limited settings—while addressing the social and structural factors driving inequities. Although anesthesia providers play a key role in patient safety and surgical outcomes, many U.S. programs still lack formal global health equity curricula [1].

Integrating this training equips residents to work in diverse and resource‐limited environments and to provide culturally responsive care. These experiences build empathy, humility, and a deeper understanding of health equity [2]. As healthcare becomes increasingly interconnected, global health expertise is essential for a resilient perioperative workforce.

Importantly for the global surgery community, anesthesia capacity is a cornerstone of safe surgical systems. The Lancet Commission on Global Surgery, as well as National Surgical, Obstetric, and Anesthesia Plans (NSOAPs), highlight that global surgery cannot advance without parallel strengthening of anesthesia education, workforce development, and leadership [3]. Therefore, structured global health equity training in anesthesiology is directly aligned with global surgical system strengthening efforts.

Amid efforts to defund health equity initiatives, integrating formal global health equity training into U.S. anesthesiology residencies has become increasingly urgent. As institutions face political and financial pressure to scale back, creating durable well‐structured curricula are essential to sustaining progress in reducing global health disparities. A clear roadmap for developing, implementing, and evaluating such programs can ensure consistency, quality, and long‐term impact. Although focused on anesthesiology, the proposed framework can serve as a scalable model for other surgical and perioperative programs aiming to build ethical, sustainable, and equity‐centered global health education pathways.

## 2 Decolonizing Global Health Education

Decolonizing global health education means examining and addressing the historical and systemic inequities reinforced by Western‐centric medical practices. In medical training, this involves elevating the perspectives of local providers in low‐ and middle‐income countries (LMICs), integrating their input into curriculum design, and ensuring they play an active role in shaping collaborations [4]. It also requires dismantling power imbalances so partnerships are equitable and truly shared.

In U.S. anesthesiology residencies, global health experiences are growing but often remain short‐term one‐sided trips with little sustainability—patterns that mirror longstanding inequities. Shifting toward equitable, bidirectional partnerships is essential for preparing clinicians to engage respectfully and effectively across diverse health systems [5]. Numerous publications [6] offer guidance on how to achieve this, including:

- *Expanding Global Health Curricula*: Incorporate courses on the history of colonial medicine and its effects on specific healthcare contexts, emphasizing the structural impacts of colonialism on LMIC healthcare systems.
- *Deemphasizing Short‐Term Engagements*: Transition from short‐term, unidirectional global health engagements to extended, reciprocal experiences that foster equitable partnerships.
- *Defining Roles and Providing Preparation*: Establish previsit assessment with clearly defined roles and responsibilities. Include site‐specific training and supervision to ensure trainees do not engage in activities beyond their level of training.
- *Building Long‐Term Collaborations*: Recruit faculty dedicated to fostering equitable sustainable global health partnerships and align institutional promotion criteria to recognize this work.
- *Bidirectionality of initiatives*: Ensure the bidirectionality of work by engaging the local providers in equal access to grant funding and/or allowing them to be on site in high income countries for learning.

By embedding these strategies into global health training programs, US anesthesiology residency education can shift from transactional engagements to drivers of transformational equity‐focused practices.

## 3 Key Domains in Global Health Competencies

The Consortium of Universities for Global Health (CUGH) Competency Subcommittee created the Global Health Education Competencies Tool Kit, outlining key domains for global health training [7]. Although not all domains apply directly to anesthesiology, they offer a strong foundation for building comprehensive relevant global health curricula. The most applicable competencies are highlighted below.

## 4 Program Design: Key Steps

The proportion of U.S. anesthesiology residencies offering global health electives is like other specialties, but programs vary widely in structure, goals, curriculum, and funding [8]. Therefore, developing a global health training program requires a clear stepwise approach. Drawing on existing and emerging initiatives, we outline key steps for creating a context‐sensitive, up‐to‐date, and decolonized global health curriculum.

Step 1: Identify Partners/Community Identification.

- Map LMIC sites for capacity for partnership.
- Build relationships with existing and current anesthesiologists (involved in or responsible for educational advancement) who can help lead, mentor, and drive priorities on the ground.
- Secure buy‐in from local stakeholders to ensure relevance and sustainability.

Step 2: Conduct a Needs Assessment.

- Use interviews, surveys, or site visits with anesthesiology providers in LMICs to understand challenges in medication access, equipment availability, and training gaps.
- Analyze the existing anesthesia landscape, including scope of practice, provider training programs, and healthcare system limitations.
- Collaborate with universities and training institutions to facilitate trainee exchanges and bidirectional hosting.
- Review available literature (e.g., WHO and WFSA) for existing material or work already done.

Step 3: Create Curriculum Material.

- Develop core lecture topics, such as:
  ◦ Principles of global health equity and key players in global health.
  ◦ Anesthesiology and the global burden of surgical disease.
  ◦ Anesthesiology practices for resource‐limited settings.
  ◦ Cultural competence and ethical considerations.
  ◦ Pain and analgesia in the global context.
- Share learning objectives and materials across institutions to streamline implementation.

Step 4: Implement teaching Deliverables.

- Deliver training using diverse methods, such as:
  ◦ Lectures, interactive workshops, case‐based discussions, and simulation‐based training.
  ◦ Virtual and in‐person mentorship programs.
  ◦ Collaborative research projects with local providers.

Step 5: Core Global Health Faculty.

- Establish a dedicated team of faculty members with global health experience.
- Assign specific responsibilities, including curriculum oversight, travel logistics, resident support, and project mentorship.

Step 6: Program Evaluation.

- Regularly assess the program's quality and impact through resident feedback and partner evaluations.
- Resident knowledge acquisition (pre/postassessments).
- Cultural competence surveys.
- Obtain LMIC partner satisfaction/feedback similar to previous studies [9, 10].
- Number of publications, QI projects, or research outputs.
- Use evaluation findings to make iterative improvements to the program.

Step 7: Sustainability Planning.

- Develop a long‐term sustainability plan that includes funding (e.g., NIH/Fogarty grants and hospital‐supported fellowships), faculty support (including protected time and promotion incentives), and material resources (such as grant writing support).
- Seek funding from grants, institutional support, or external donors.
- Build partnerships with global health organizations for additional support and expertise.

Step 8: Research, Quality Improvement, and Mentorship Opportunities.

- Encourage residents to engage in global health research from study design to data collection and analysis.
- Pair residents with experienced global health mentors to guide their professional development.

## 5 Existing Global Health in Anesthesia Training Programs

There are several US anesthesiology residency training programs that have aligned their curriculum to best match the CUGH toolkit (Table 1); however, this list is not exhaustive. This table was created utilizing information provided on program websites and author knowledge.

**TABLE 1:** Examples of anesthesiology residency programs that have aligned curriculum to CUGH toolkit.

Institution	Key curriculum components	Core faculty	Resident training programs	Rotations	Funding sources	Partner sites
UCSF	Focus on health disparities local and international, anesthesia and surgical disease burden, pain, and analgesia in the global context, anesthesia in resource limited settings with an emphasis on research and QI improvement projects.	15 core faculty (global anesthesia and surgery)	CA‐3 onsite rotation up to 3 months of dedicated research and project time either locally or internationally. Option to participate in the health equity curriculum. Option for global health equity fellowship with a 50% clinical commitment and 50% commitment to research, policy, and education	Operation rainbow and international electives	Resident Clinical and Translational Research Funding (RRF) ‐ up $5000	Busitema University and Makerere University in Uganda
Stanford	Global anesthesia advanced clinical experience (ACE), equity seminar series	9 faculty	Global anesthesia pathway	1‐week service trips and 4‐week teaching rotations	Internal anesthesia research grant, center for innovation in global health seed grant, McCormick and Gabilian faculty awards, awards through the king center for global development, and the Rosenkranz Prize	University of Rwanda, Foundation for African Medicine and Education (FAME) in Karatu, Tanzania. Hue University Medical and Pharmacy (HUMP) in Vietnam. Global health ‐ professional equity and exchange program (GH‐PEEP), which brings visiting observers from international partnership sites to interact locally with Stanford's faculty, trainees, and students
Vanderbilt	Global health pathway, mentorship, and journal clubs	7 faculty	Global health residents starting intern/CA‐1 year.	1 month in CA‐2 years and optional additional month in CA‐3 years	Vanderbilt international anesthesia funding preference, ImPACT (improving perioperative and anesthesia care) Africa grant‐ a $3 million dollar grant from GE foundation to fund international medical education and research in Kenya and other low resource settings.	AIC Kijabe hospital in Kenya, Mozambique, Ethiopia, Tanzania
UCLA	Global health equity and perioperative disparities Global surgical disease burden and anesthesia Pain and analgesia in the global context Anesthesia in resource‐limited settings Cultural humility and ethical considerations	6 faculty	Residents join the pathway as CA‐1, max of 4 residents/year, and clinical and research rotation as CA‐3s	4 weeks	100% ‐ departmental funding + internal research grants	Tikur Anbessa Specialized Hospital, Addis Ababa Ethiopia
Weill cornell	Approaching global health via three programmatic spheres: Education, research, and implementation programming; involves undergraduates, medical students, anesthesiology residents, and anesthesiology faculty; educational component employs a biopsychosocial, bidirectional approach, and includes dedicated didactic sessions focused on global health topics for anesthesiology residents; and a 6‐session curriculum for those residents and faculty participating in a global health elective	4 core faculty	2–4 weeks teaching rotations in CA‐3 years Option for residency global health track Option for global health fellowship	2–4 weeks	Departmental, ASA charitable foundation global health overseas training program; research funding from the foundation for anesthesia education and research (FAER)	University of Rwanda and University of Global Health Equity in Rwanda; Sri Guru Ram Das Institute of Medical Sciences and Research in India

## 6 Conclusion

Creating a global health equity training program for U.S. anesthesiology residencies requires commitment, collaboration, and a clear equity‐focused vision. By following this roadmap, programs can build a sustainable curriculum that prepares interested residents to navigate complex global health challenges locally and abroad. Prioritizing a decolonized inclusive approach promotes culturally responsive care and equitable partnerships. This is an opportunity to train anesthesiologists who are both technically skilled and deeply committed to improving health outcomes worldwide.

## Author Contributions

**Betelehem M. Asnake:** conceptualization, writing – original draft, writing – review and editing. **Maziar M Nourian:** conceptualization, writing – original draft, writing – review and editing. **Ana M. Crawford:** supervision. **Bantayehu Sileshi:** supervision. **Sheida Tabaie:** writing– review and editing.

## Funding

The authors have nothing to report.

## Conflicts of Interest

The authors declare no conflicts of interest.

## Data Availability

Data sharing is not applicable to this article as no datasets were generated or analyzed during the current study.

## References

[1] The ACGME and Global Health , “International PGME Partnerships to Support Training Physicians into the Future,” Accessed, September 10, 2025, https://www.acgme.org/newsroom/blog/2024/the‐acgme‐and‐global‐health‐international‐pgme‐partnerships‐to‐support‐training‐physicians‐into‐the‐future/.

[2] E. Wollner , T. Law , K. Sullivan , and M. S. Lipnick , “Why Every Anesthesia Trainee Should Receive Global Health Equity Education,” Canadian Journal of Anesthesia/Journal canadien d'anesthésie 67, no. 8 (2020): 924–935, 10.1007/s12630-020-01715-3. 32483743

[3] L. Roa , D. T. Jumbam , E. Makasa , and J. G. Meara , “Global Surgery and the Sustainable Development Goals,” British Journal of Surgery 106, no. 2 (2019): e44–e52, 10.1002/bjs.11044. 30620060

[4] Q. G. Eichbaum , L. V. Adams , J. Evert , M. J. Ho , I. A. Semali , and S. C. van Schalkwyk , “Decolonizing Global Health Education: Rethinking Institutional Partnerships and Approaches,” Academic Medicine 96, no. 3 (2021): 329–335, 10.1097/ACM.0000000000003473. 32349015

[5] M. Stoltenberg , N. Rumas , and K. Parsi , “Global Health and Service Learning: Lessons Learned at US Medical Schools,” Medical Education Online 17, no. 1 (2012): 18848, 10.3402/meo.v17i0.18848. PMC3401288 22826631

[6] D. L. Garba , M. C. Stankey , A. Jayaram , and B. L. Hedt‐Gauthier , “How Do We Decolonize Global Health in Medical Education?,” Annals of Global Health 87, no. 1 (2021): 29, 10.5334/aogh.3220. 33816134 PMC7996454

[7] Global Health Competencies Toolkit , “Consortium of Universities for Global Health,” Accessed, September 10, 2025, https://www.cugh.org/online‐tools/competencies‐toolkit/.

[8] G. Kaur , S. Tabaie , J. Brar , V. Tangel , and K. O. Pryor , “Global Health Education in United States Anesthesiology Residency Programs: A Survey of Resident Opportunities and Program Director Attitudes,” BMC Medical Education 17, no. 1 (2017): 215, 10.1186/s12909-017-1056-3. 29145835 PMC5689206

[9] E. Amick , F. Sharmin , S. Bucher , and B. W. Henry , “Low‐ and Middle‐Income Country Perceptions of Global Health Engagements: A Scoping Review,” Global Journal of Health Science 16, no. 3 (2024): p35, 10.5539/gjhs.v16n3p35.

[10] K. N. Krakauer , L. Y. Wong , J. Tobias , et al., “Evaluating Global Surgery Partnerships From Low and Middle Income Country Perspectives,” Journal of Surgical Research 296 (2024): 681–688, 10.1016/j.jss.2024.01.040. 38364695

